# Supplementary figures and images for: Listeria monocytogenes InlP interacts with afadin and facilitates basement membrane crossing
Source: PLoS Pathog. 2018 May 30;14(5):e1007094. doi: 10.1371/journal.ppat.1007094 (PMC6044554; doi:10.1371/journal.ppat.1007094)

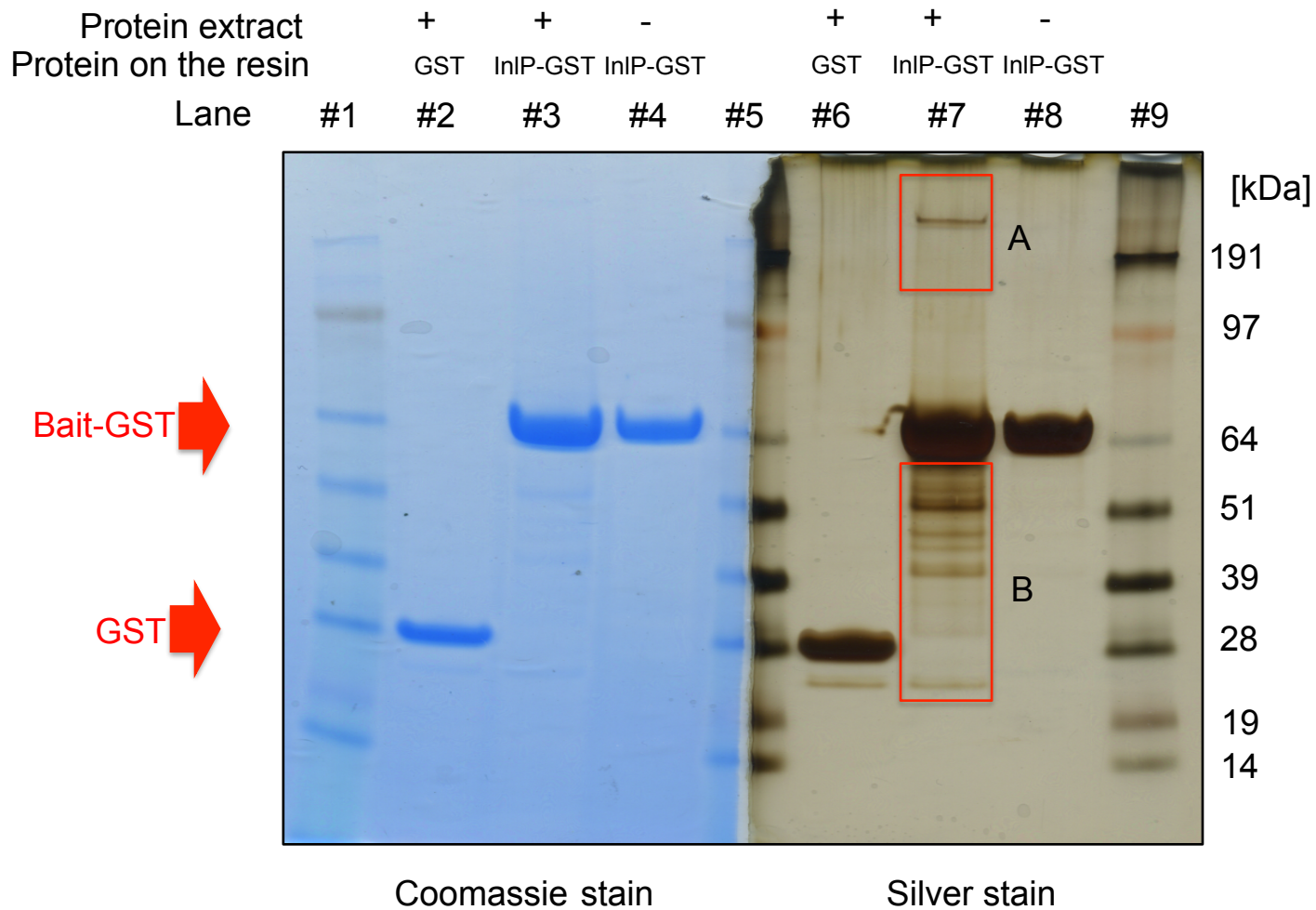

**S1 Figure**

Supplement: S1 Fig — GST protein alone and InlP-GST fusion protein bound to glutathione-Sepharose resin were used as bait for pull-down experiments with (+) or without (-) protein extracts from human placenta. Shown on the left is Coomassie blue staining of each fraction, with the most abundant band in each lane representing the bait protein. Shown on the right is silver staining of each fraction, with the most abundant band in each lane representing the bait protein. The red boxes labeled A and B show the two fractions extracted from gel and analyzed by mass spectrometry (S1 Table). (PDF) [file ppat.1007094.s001.pdf]

A

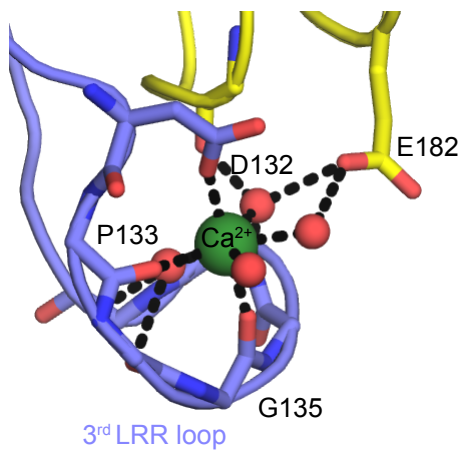

B

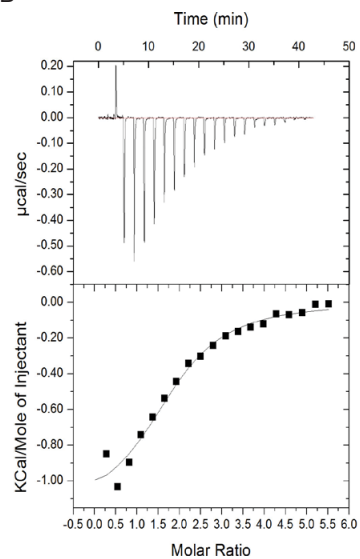

S2 Figure

Supplement: S2 Fig — (A) Schematic view of the interaction between the Ca2+ with the 3rd LRR loop of InlP. The amino acids D132 and E182, which are involved in the interaction, are shown as sticks, water molecules as red spheres, and hydrogen bonds as dashed lines. (B) Isothermal titration calorimetry results show Ca2+ binding to InlP. The isotherm was fit by a one site binding model (N = 1.8 ± 0.1 site, K = 2.6E4 ± 6.5E3 M-1, ΔH = -1176 ± 90.2 cal/mol, ΔS = 16.3 cal/mol/deg). (PDF) [file ppat.1007094.s002.pdf]

A

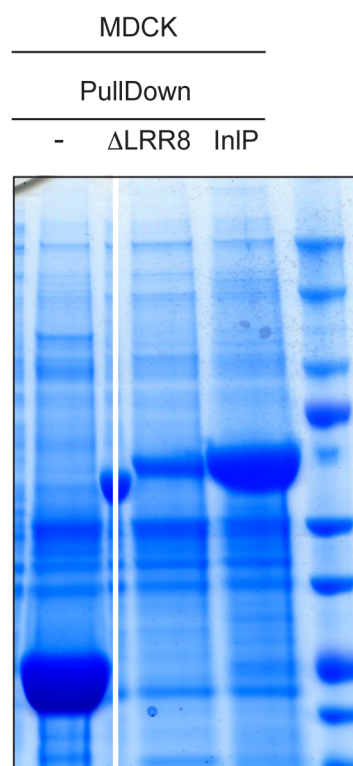

B

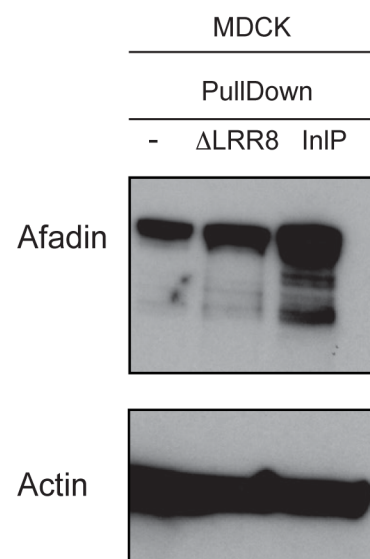

**S3 Figure**

Supplement: S3 Fig — InlP-afadin binding pull-down experiments with ΔLRR8 mutant. GST protein alone (-), InlPΔLRR8-GST fusion protein (ΔLRR8), or InlP-GST (InlP) bound to glutathione-sepharose resin were used as bait for pull-down experiments with protein extracts from MDCK cell line. (A) Coomassie blue staining of each fraction, with the most abundant band in each lane representing the bait protein. (B) Data shown are a western blot analysis using anti-afadin antibodies. Actin was used as loading control. (PDF) [file ppat.1007094.s003.pdf]

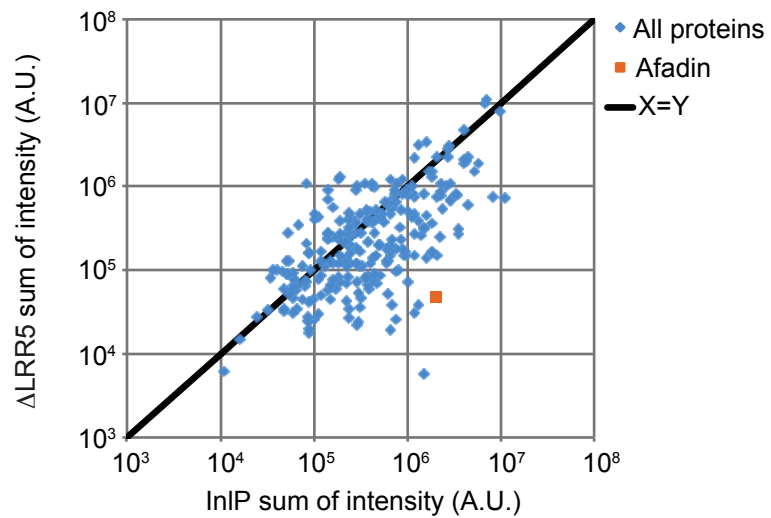

**S4 Figure**

Supplement: S4 Fig — Scatter plot of intensities of InlP-GST versus ΔLRR5-GST binding proteins coming from MDCK cell cultures extracts. Plot shows the sum of intensities (A.U.) of the proteins identified through mass spectrometry using InlP-GST or InlPΔLRR5-GST as baits to identify host binding partners in the MDCK extracts. Blue diamonds show all the proteins apart from afadin, which is indicated as orange square. Black line shows X = Y. Filters applied to the data are discussed in the Methods section. (PDF) [file ppat.1007094.s004.pdf]

A

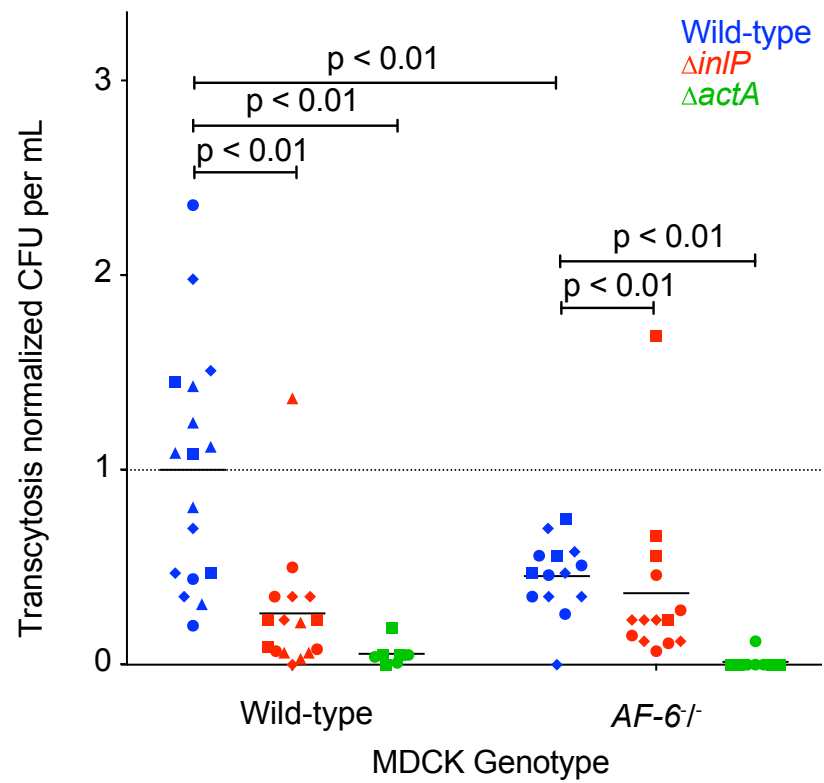

B

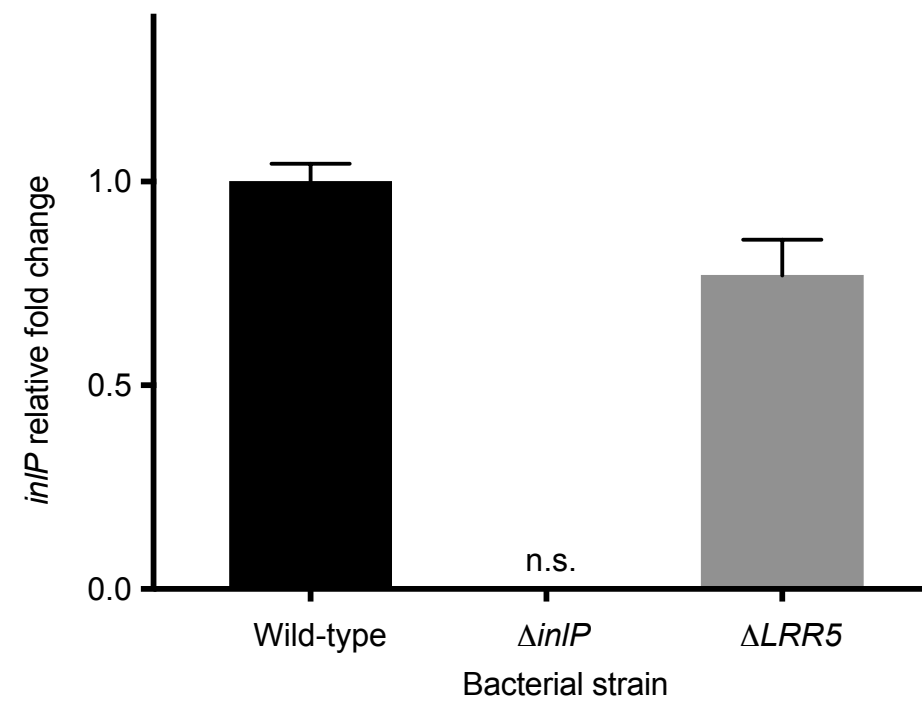

S5 Figure

Supplement: S5 Fig — (A) Amount of transcytosis by wild-type (blue), ΔinlP (red), and ΔactA (green) L. monocytogenes through MDCK and MDCK AF-6-/- monolayers. Data were normalized to 1 for MDCK cells infected with wild-type L. monocytogenes for each experiment, and pooled from four independent experiments (MDCK cells) or three independent experiments (MDCK AF-6-/- cells). Each experiment is depicted by different symbols. (B) Quantification through RT-qPCR of InlP expression for wild-type L.monocytogenes, ΔinlP and inlPΔlrr5 mutants grown in BHI liquid media. Ribosomal prokaryotic RNA (16S) was used for normalization. Relative fold change in gene expression with respect to wild-type L.monocytogenes. N = 3–6 replicates are shown for each group. (PDF) [file ppat.1007094.s005.pdf]
