# Supplementary material for: Listeria monocytogenes InlP interacts with afadin and facilitates basement membrane crossing
Source: PLoS Pathog. 2018 May 30;14(5):e1007094. doi: 10.1371/journal.ppat.1007094 (PMC6044554; doi:10.1371/journal.ppat.1007094)
Supplement: S1 Table — (PDF) [file ppat.1007094.s006.pdf]

**S1 Table. Mass spectrometry data from pull-downs of InlP-GST.**

Proteins identified with more than 10 unique peptides from human placental extract pull-downs are outlined below and ordered based on descending molecular weight. Black and blue gels correspond to sections A and B respectively (see Supplementary Figure 1). Afadin is indicated in red. Columns 3-5 correspond to pull-downs from placental extract with InlP-GST. Columns 6-7 show the corresponding total intensities from mass spectrometry data using InlP-GST and GST alone as baits to identify host binding partners in extracts from MDCK cell cultures.

| | | Placental extract pull-down | | | MDCK extract pull-down | |
|---|---|---|---|---|---|---|
| **Gene name** | **Molecular weight (kDa)** | **Unique peptides** | **Total peptides** | **Total Intensity InlP-GST** | **Total Intensity InlP-GST** | **Total Intensity GST** |
| | | | | | | |
| AHNAK | 628.70 | 54 | 67 | $1.2x10^5$ | $3.3x10^6$ | $1.6x10^6$ |
| DYNC1H1 | 532.07 | 39 | 54 | $7.6x10^5$ | $1.7x10^6$ | $6x10^4$ |
| COL6A3* | 343.66 | 24 | 27 | $2.5x10^5$ | 0 | 0 |
| FLNA | 280.56 | 79 | 124 | $1.3x10^6$ | $2.9x10^6$ | $9.9x10^5$ |
| FLNB | 277.99 | 29 | 38 | $5.7x10^5$ | $6.4x10^6$ | $3x10^6$ |
| TPR* | 267.29 | 29 | 39 | $6.3x10^5$ | 0 | 0 |
| MYH9 | 226.39 | 14 | 17 | $1.2x10^5$ | $4.1x10^7$ | $1.2x10^7$ |
| AF6 | 206.68 | 18 | 23 | $5.3x10^5$ | $2x10^6$ | 0 |
| COL14A1* | 193.51 | 11 | 12 | $1.6x10^5$ | 0 | 0 |
| CLTC | 191.49 | 20 | 26 | $4.0x10^5$ | $9.8x10^6$ | $9x10^6$ |
| IQGAP1 | 189.13 | 25 | 31 | $3.9x10^5$ | $9.6x10^6$ | $1.9x10^5$ |
| TNKS1BP1 | 181.69 | 15 | 19 | $1.5x10^5$ | $7.9x10^4$ | 0 |
| FN1* | 181.23 | 11 | 14 | $3.4x10^5$ | 0 | 0 |
| KIF5B | 109.62 | 13 | 15 | $2.6x10^5$ | $4.3x10^6$ | $1.9x10^5$ |
| ACADVL* | 70.39 | 12 | 16 | $1.6x10^6$ | 0 | 0 |
| CALD1* | 62.66 | 13 | 27 | $3.7x10^6$ | 0 | 0 |
| ANXA1 | 54.36 | 12 | 23 | $1.2x10^7$ | $2x10^8$ | $2.5x10^7$ |
| VIM | 53.62 | 16 | 27 | $5.7x10^6$ | $3x10^7$ | $1x10^7$ |
| ANXA2 | 38.58 | 18 | 38 | $2.3x10^7$ | $2x10^6$ | $x10^6$ |
| ANXA5 | 35.91 | 11 | 14 | $7.9x10^6$ | $7.7x10^5$ | $6.5x10^6$ |
| GSTP1* | 23.35 | 11 | 24 | $3.2x10^7$ | 0 | 0 |

* Asterisk denotes that no peptides were identified in extracts from MDCK cell cultures.
