# Supplementary material for: Listeria monocytogenes InlP interacts with afadin and facilitates basement membrane crossing
Source: PLoS Pathog. 2018 May 30;14(5):e1007094. doi: 10.1371/journal.ppat.1007094 (PMC6044554; doi:10.1371/journal.ppat.1007094)
Supplement: S2 Table — (PDF) [file ppat.1007094.s007.pdf]

S2 Table. Yeast two-hybrid screening of human placenta library
Results summary listing the protein partners identified (gene name), the number of independent clones and the global PBS [a]. Ordering is performed based on global PBS and then on number of clones. Afadin is indicated in red.

Gene name	Number of independent clones	Global PBS [a]
RPS8	26	A
RBM5	14	A
TSHZ1	12	A
RPL5	9	A
AEBP1	7	A
NAB2	6	A
THAP3	6	A
NAB1	4	A
DNMT3A	5	B
SAFB	5	B
ZNF653	5	B
EXOSC3	4	B
RPL7A	4	B
AF6	3	B
FLJ22329	3	B
SP3	3	B
ZNF653	5	B
ZNF6	3	C
CDC27	2	C
EPS8L2	2	C
PHF23	2	C
ZNF283	2	C

[a]Global Predicted Biological Score (PBS). Confidence score of each interaction. A: very high confidence interaction; B: high confidence in the interaction; and C: good confidence in the interaction.
