# Supplementary material for: Listeria monocytogenes InlP interacts with afadin and facilitates basement membrane crossing
Source: PLoS Pathog. 2018 May 30;14(5):e1007094. doi: 10.1371/journal.ppat.1007094 (PMC6044554; doi:10.1371/journal.ppat.1007094)
Supplement: S3 Table — (PDF) [file ppat.1007094.s008.pdf]

S3 Table. Crystallographic Table

Data Collection and Refinement Statistics[a]

	**InlP**	**Lmo2027**
PDB code	5HL3	5KZS
Data Collection		
Resolution range, Å	30.00-1.40 (1.42-1.40)	30.00-2.30 (2.34-2.30)
Space group	$P4_12_12$	I_4
Unit cell dimensions		
a,b,c, Å	a & b= 72.9 c = 179.5	a & b = 157.8 c = 36.3
α, β, γ, degrees	α, β, γ = 90	α, β, γ = 90
Completeness, %	99.8 (100)	98.6 (99.6)
No. reflections	180423 (9031)	20005 (1429)
Redundancy	45.6 (5.3)	6.2 (4.5)
$\langle I/\sigma(I)\rangle$	30.2 (2.6)	79.7 (2.0)
R_{merge}, %	7.9 (65.3)	9.0 (57.1)
Refinement statistics		
R-factor (R_{work}/R_{free})[b]	16.0/17.6	22.8/27.9
No. atoms		
Protein	2935	2377
Waters	828	26
Ca^{2+}	8	n/a
R.M.S. deviations		
Bond lengths, Å	0.011	0.009
Bond angles, degrees	1.51	1.47
Ramachandran analysis		
Favored regions, %	96	95
Allowed regions, %	100	99
Disallowed regions, %	0	1

[a] Highest resolution shell in parenthesis.

[b] Definition of R_{work}, R_{free}: $R=\Sigma_{hkl}\left||F_{obs}|-|F_{calc}|\right| / \Sigma_{hkl}|F_{obs}|$, where *hkl* are the reflection indices used in refinement for R_{work}**, and 5% not used in refinement for** R_{free}. F_{obs} and F_{calc} are structure factors deduced from measured intensities or calculated from the model, respectively.
